# Genomic Changes of Chagas Disease Vector, South America

**DOI:** 10.3201/eid1003.020812

**Published:** 2004-03

**Authors:** Francisco Panzera, Jean Pierre Dujardin, Paula Nicolini, María Noel Caraccio, Virginia Rose, Tatiana Tellez, Hernán Bermúdez, María Dolores Bargues, Santiago Mas-Coma, José Enrique O’Connor, Ruben Pérez

**Affiliations:** * Universidad Mayor de la República, Montevideo, Uruguay; † Institut de Recherche pour le Développement, Montpellier, France; ‡ Universidad Mayor de San Simón, Cochabamba, Bolivia; § Universidad de Valencia, Valencia, Spain

**Keywords:** Chagas disease, Triatominae, holocentric chromosomes, heterochromatin, genome size, *Triatoma infestans*, geographic polymorphism, flow cytometry

## Abstract

We analyzed the main karyologic changes that have occurred during the dispersion of *Triatoma infestans*, the main vector of Chagas disease. We identified two allopatric groups, named Andean and non-Andean. The Andean specimens present C-heterochromatic blocks in most of their 22 chromosomes, whereas non-Andean specimens have only 4–7 autosomes with C-banding. These heterochromatin differences are the likely cause of a striking DNA content variation (approximately 30%) between Andean and non-Andean insects. Our study, together with previous historical and genetic data, suggests that *T. infestans* was originally a sylvatic species, with large quantities of DNA and heterochromatin, inhabiting the Andean region of Bolivia. However, the spread of domestic *T. infestans* throughout the non-Andean regions only involved insects with an important reduction of heterochromatin and DNA amounts. We propose that heterochromatin and DNA variation mainly reflected adaptive genomic changes that contribute to the ability of *T. infestans* to survive, reproduce, and disperse in different environments.

American trypanosomiasis or Chagas disease is well recognized as the most serious human parasitic disease of the Americas in terms of its social and economic impact (*1*). This disease is caused by the flagellate protozoan *Trypanosoma cruzi,* and it is transmitted by blood-sucking insects of the subfamily Triatominae (Hemiptera, *Reduviidae*). There is no vaccine against *T. cruzi*; therefore, disease control relies on eliminating domestic vector populations by spraying infested houses with residual insecticides.

The epidemiologic importance of Chagas disease vectors largely depends on the vectors’ spreading ability and adaptation to domestic habitats. Therefore, studies on the changes that have taken place in such domestication and geographic expansion may contribute to understanding the basic process by which some species of Triatominae invade new habitats and colonize human habitations. These analyses are fundamental in the design of control campaigns because their results will help determine the most appropiate strategy for insecticide application. Knowledge of the genetic structure of insect populations (including the evaluation of gene flow between domestic and sylvatic populations), as well as their domestication and spreading capabilities, are essential tools for effective vector control (*2*).

*Triatoma infestans* represents the best example of spreading and adaptation to domicilies observed in a triatomine species. This species is the main and widespread vector in South America, responsible for about half of the 12 million cases of Chagas disease reported worldwide. Although its distribution is now being substantially reduced by large-scale control interventions within the Southern Cone Initiative, launched in 1991 by five South American countries (*1*), its distribution in the mid-1980s was very wide, including vast regions of Argentina, Bolivia, Brazil, Chile, Paraguay, southern Peru, and Uruguay (Figure 1). *T. infestans* is found almost exclusively in domestic and peridomestic environments, occupying cracks and crevices in rural dwellings and domestic animal enclosures. The presence of this species in sylvatic habitats (rock piles in association with wild guinea pigs) has only been confirmed in the Andean valleys of Cochabamba and Sucre in Bolivia (*3*–*5*). This finding, together with historical reconstruction (*6*) and genetic analyses (*7*), suggests that central Bolivia may be the site of origin and dispersal of *T. infestans* throughout South America.

**Figure 1:**
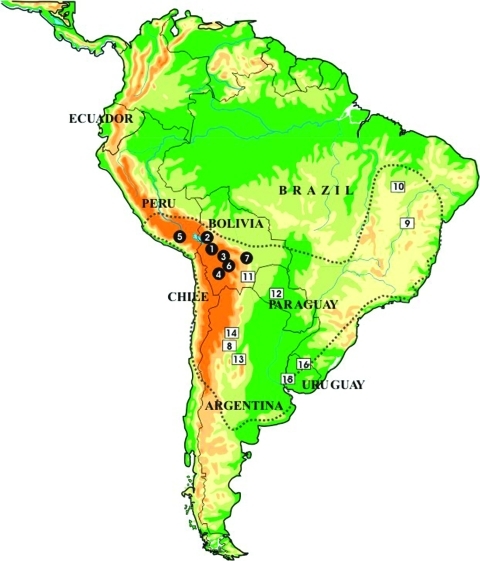
Location of the collection sites of individual *Triatoma infestans* analyzed in this study. Dotted lines indicate *T. infestans* distribution during the 1980s. Full circles indicate Andean samples. Open squares indicate non-Andean samples. (See number identification of each population in Tables 1–3.)

One important approach used to establish genetic variation in *T. infestans* is cytogenetic analysis. The diploid chromosome number of *T. infestans* is 22*,* including 10 pairs of autosomes and 1 pair of sex chromosomes (XY in males, XX in females) (*8*). The three large autosomal pairs and the Y chromosome present C-heterochromatic blocks (*9*). Based on a great variation in the quantity and position of these C-heterochromatic regions, an extensive polymorphism in natural populations from Uruguay has been described (*10*). This variation in the three large autosomal pairs was also described in laboratory-reared specimens from Brazil, Paraguay, Argentina, and Chile (*11*,*12*).

We present an extensive analysis, including specimens from several countries (Figure 1). Using flow cytometry for DNA quantification and C-banding technique, we have determined the karyologic changes that have occurred during the dispersal of *T. infestans*. This analysis has allowed us to identify two very different chromosomal groups and to discuss the role of heterochromatin and genome size variation in the karyologic evolution of *T. infestans*.

## Materials and Methods

### Material Analyzed

A total of 209 *T. infestans* specimens from natural populations were examined by C-banding (Tables 1 and 2, Figure 1). Currently, several of these populations, particularly from Uruguay and Brazil, have disappeared as a result of intensive control interventions. Five male specimens of the experimental progeny obtained by crossing insects from Brazil and Bolivia (Cochabamba Valley) were also examined. Flow cytometric analysis of DNA content was performed in 42 male insects. Student *t* test was used for statistical analysis of the results obtained by C-banding and flow cytometry; p < 0.001 was considered significant.

**Table 1:** Analyzed material of *Triatoma infestans* classified by procedence, biogeographic region, altitude, number of specimens analyzed (n) with C-banding, and number of autosomes with C-bands (mean and standard deviation)^a^

Country	Department, province, locality; habitat^b^; y collected	Biogeographic region^c^	Altitude (m)	N (M,F)^d^	No. of C-autosomes mean and SD	
Bolivia	La Paz, Murillo, Palomar. D. 1997	Andes [1]	3,000	3 M	18.00 + 2.00	
Bolivia	La Paz, La Paz, Río Abajo. D. 1997	Andes [2]	2,900	16 M	17.00 + 1.03	
Bolivia	Cochabamba, Esteban Arze, Jamach´Uma. D. 1997	Andes [3]	2,700	15 M, 3 F	15.72 + 1.49	
Bolivia	Cochabamba, Esteban Arze, Jamach´Uma. S. 1997	Andes [3]	2,700	7 M, 2 F	16.00 + 0.87	
Bolivia	Chuquisaca, Yamparaez, Uyuní. D. 1997	Andes [4]	2,542	9 M	17.44 + 0.88	
Peru	Arequipa , Arequipa city. D. 1997	Andes [5]	2,336	8 M	16.63 + 1.06	
Bolivia	Cochabamba, Campero, Peña Colorada. D. 1997	Andes [6]	1,890	3 M	16.00 + 0.00	
Bolivia	Santa Cruz, Florida, Pampa Grande. D. 1997	Andes [7]	1,250	4 M	16.75 + 0.50	
Argentine	La Rioja, Anillaco. P. 1997	Austral Chaco [8]	1,400	5 M	6.20 + 0.45	
Brazil	Bahia, Paratinga. D. 1995	Caatinga [9]	500	9 M	6.00 + 0.00	
Brazil	Piaiu, Caracol. D. 1996	Caatinga [10]	450	6 M, 8 F	6.00 + 0.00	
Bolivia	Santa Cruz, Cordillera, Izozog. D. 1997	Boreal Chaco [11]	350	2 M	7.00 + 0.00	
Bolivia	Santa Cruz, Cordillera, Izozog. S. “Dark morphs.” 1997	Boreal Chaco [11]	350	8 M	6.00 + 0.00	
Paraguay	Chaco, Río Negro. D. 1997	Boreal Chaco [12]	350	6 M, 3 F	6.33 + 0.50	
Argentine	Córdoba, Cruz del Eje, Los Leones. D & P. 2000	Austral Chaco [13]	250	12 M	5.17 + 0.58	
Argentine	Santiago del Estero, Moreno, San Pablo. P. 1999	Austral Chaco [14]	200	7 M, 3 F	5.50 + 0.85	
Uruguay	Several populations from Southern and Northern. D & P. 1988–1995	Pampeana [15,16]	0–200	44 M, 26 F	5.99 + 0.12	

^a^All specimens came from natural populations. Statistically significant differences (p < 0.001) were detected in the number of C-autosomes between Andean (16.54 + 1.29) and non-Andean (5.93 + 0.45) grouped samples. ^b^P, peridomiciliary; D, domiciliary; S, sylvatic. ^c^Numbers in brackets refer to the location of the populations in Figure 1 ^d^M, males; F, females.

**Table 2:** C-banding patterns observed in the three largest autosomal pairs of *Tiratoma infestans* from the non-Andean populations analyzed^a^

C-banding pattern	Argentina (Austral Chaco) [8][13][14]^b^	Bolivia and Paraguay (Boreal Chaco) [11,12]	Uruguay (Pampeana) [15,16]	Brazil (Caatinga) [9,10]	Total specimens	
BB BB BB	-	1	-	-	1	
BB BB AB	1	7	6	-	14	
BB BB AA	1	3	43	21	68	
BB BB AC	1	-	1	-	2	
BB AB AA	4	-	16	1	21	
BB AA AA	4	1^a^	4	1	10	
BB AA AC	3	-	-	-	3	
BB AB AC	2	-	-	-	2	
BB AB CC	2	-	-	-	2	
BB AA CC	1	-	-	-	1	
AB BB AA	1	-	-	-	1	
AB AB AA	2	-	-	-	2	
AB AB AC	3	-	-	-	3	
AB AA AB	1	-	-	-	1	
AB AA AA	1	3^c^	-	-	4	
AA AA AA	-	4^c^	-	-	4	
Total	27	19	70	23	139	

^a^The population more near the Andean region of Bolivia and Peru, e.g., the Austral Chaco region of Argentine, appeared very variable both in the number of C-banded autosomes and in the karyomorphs observed. By contrast, the samples farthest away from the Andean region, e.g., Brazilian Caatinga populations, were the most homogeneous, almost always exhibiting the same C-karyomorphs (BB BB AA). ^b^Numbers in brackets refer to the location of the populations in Figure 1. ^c^Sylvatic (dark morphs).

### Chromosome Preparations and Banding Procedures

Gonads (testes and ovaries) from adult insects (occasionally fifth-stage nymphs) were removed, fixed in ethanol‑acetic acid (3:1), and stored at –20°C. C-banding treatment was carried out on air-dried squash, as previously described (*13*).

The C-banding pattern for each specimen was determined by analyzing at least 10 cells. In males, both mitotic (spermatogonial prometaphase) and meiotic (metaphase I or II) plates were observed. For females, only oogonial prometaphases were studied because no meiotic stages can be detected.

**Figure 2:**
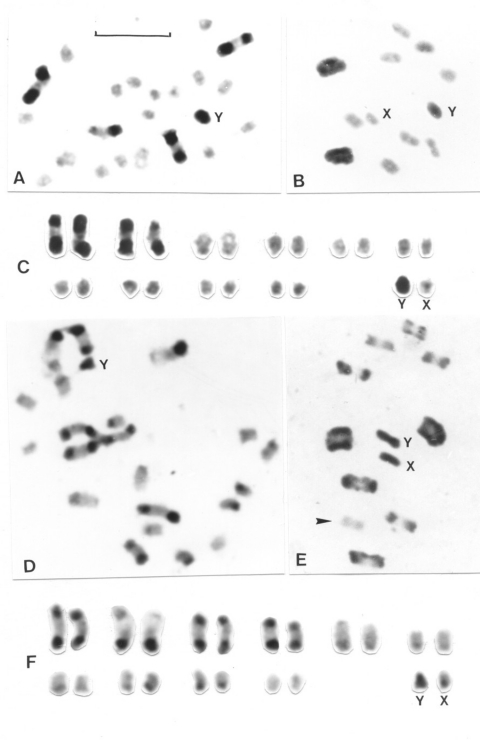
Representative C-banding patterns observed in male *Triatoma infestans*: 2n = 22 (20 autosomes plus XY in males/ XX females) coming from non-Andean (A-C) and Andean regions (D-F). Scale bar = 10 μm. A: Spermatogonial mitotic prometaphase. This specimen from Argentina presents the lowest number of C-banded autosomes (four chromosomes). B: First meiotic metaphase. Only two heterochromatic bivalents, formed by the pairing of the four C-banded autosomes showed in 2A, are observed. The Y (heterochromatic) and X (euchromatic) chromosomes appear as univalents, as typically observed in hemipteran insects. C: Karyotype obtained from 2A. Heterochromatic C-bands are clearly detected in four autosomes and in the Y sex chromosome. D: Mitotic prometaphase in male specimen from Andean Bolivia. Almost all chromosomes present C-bands in one or both chromosomal ends. E: First meiotic metaphase of the same insect shown in 2D. All bivalents except one (arrowhead) are formed by chromosome with C-bands. As observed in other hemipterans, the bivalents form a ring with the univalent sex chromosomes (X and Y) in the center. F: Karyotype obtained from 2D. Chromosome size and C-banding pattern are clearly different from those observed in 2C. Heterochromatic blocks are localized in most autosomes and in both sex chromosomes.

The identification of each chromosomal pair was based on size differences and on the analysis of the meiotic configurations. Each pattern can be assigned to the corresponding chromosomal pair only when C-heterochromatin is present in three or four autosomal pairs. To describe the different C-banding patterns, three autosomal morphs, denoted A, B, and C, were recognized on the basis of previous reports (*10*,*12*) (Figure 2): A morph (a subterminal C-heterochromatic block is present at one chromosomal end; the other end is euchromatic or has a very small C-band); B morph (C-heterochromatic blocks are clearly present at both chromosomal ends); and C morph (the chromosome is totally euchromatic or has a very small C-band).

We estimated the relative length of the C-heterochromatin in the total length of the autosomal complement. At least three specimens from each population were analyzed. For each specimen, three to five photographs of the gonial metaphase plate were digitized and quantified by means of appropriate software (IPP plus, Media Cybernetics, Carlsbad, CA).

### Measuring Genome Size by Flow Cytometry

To establish the haploid genome size, we used flow cytometry to measure nuclear DNA content in gonad cells from 42 male insects (Table 3) previously fixed in ethanol-acetic acid (3:1). Gonads from fixed insects were excised and deposited on excavated glass slides. A few drops of hypotonic DNA-staining buffer (HDSB), containing 0.1% trisodium citrate, 0.1% Triton X-100, 100 μg/mL RNAase A, and 50 μg/mL propidium iodide) were added to cover the tissue. Gonads were then minced by using scalpel blades until homogeneous slurries were obtained. These were transferred with a Pasteur pipette to 5-mL polypropylene tubes, with the glass slides washed with additional HDSB to obtain a final volume of 2 mL. The suspensions were then incubated for 30 min at 37°C in the dark with occasional vortexing of the tubes. Immediately before flow cytometric analysis, suspensions were filtered through 60-μm nylon mesh. To evaluate absolute DNA contents, we used as reference the Normal DNA Index (Coulter Cytometry, PN 6699500). This reagent consists of normal human lymphocytes fixed in ethanol: acetic acid and is a standard for the human lymphocyte genome size (2 C= 6.436 pg). All measurements were performed on an EPICS XL-MCL flow cytometer (Coulter Electronics, Hialeah, FL) with an air-cooled argon-ion laser tuned at 488 nm and 15 mW. Propidium fluorescence (FL3), proportional to DNA content, was collected through a 650-nm DL dichroic filter plus a 625-nm BP band-pass filter. Forward and side scatter signals were used for morphologic assessment of the samples. Cell aggregates and coincident cells were excluded by analysis of the relationship between FL3 integral and peak signals. DNA content in single cells was determined from FL3 linear histograms. The absolute DNA amount was calculated from the ratio of the mean channel of the insect haploid G0 peak to the mean channel of the human lymphocyte diploid G0 peak. To standardize the measurements, the flow cytometer was calibrated every day with standard FlowSet fluorescent microspheres (Coulter Cytometry), and replicate samples of Normal DNA Index were run with every batch of insect gonad cells.

**Table 3:** Haploid DNA contents (C-value) expressed in pg (mean and standard deviation), measured by flow cytometry, in 42 *T. infestans* specimens from different populations^a^

Origin	Population analyzed^b^	N	Haploid DNA content mean and SD (pg)^c^
Bolivia (Andean)	Jamach’Uma. D. [3]	4	1. 842 + 0.201
Bolivia (Andean)	Jamach’Uma. S. [3]	4	1. 835 + 0.140
Bolivia (Andean)	Río Abajo. D. [2]	4	1.799 + 0.140
Paraguay (non-Andean)	Chaco. D. [12]	4	1.494 + 0.170
Brazil (non-Andean)	Caracol and Paratinga. D. [9,10]	3	1.420 + 0.041
Uruguay (non-Andean)	Northern populations. P. D. [16]	13	1.414 + 0.106
Argentine (non-Andean)	Cruz del Eje and Moreno. P. D. [13,14]	6	1.352 + 0.094
Bolivia (non-Andean)	Santa Cruz. S. Dark morphs [11]	4	1.320 + 0.046

^a^n, number of specimens analyzed; P, peridomiciliary; D, domiciliary; S, sylvatic. ^b^Numbers in brackets refer to the location of the populations in Figure 1. ^c^Significant differences (p < 0.001) were detected in C-values between Andean (1.825 + 0.149) and non-Andean (1.401 + 0.111) grouped samples.

## Results

All *T. infestans* specimens had the same diploid chromosome number (2n = 22), constituted by 20 autosomes and two sex chromosomes (XY in the males and XX in the females). C-heterochromatic blocks were usually located in terminal and subterminal positions. Interstitial C-bands were exceptional. Each specimen exhibited a specific C-banding pattern, without intraindividual variation. Tables 1 and 2 and Figures 1 and 2 summarize the large variability observed in the C-banding karyotype of *T. infestans* from different localities. All populations showed variation in the number and/or the position of C-bands, allowing us to differentiate two clearly distinct groups.

Group 1 includes insects from all Andean populations from Bolivia and Peru (70 specimens). The number of autosomes with C-blocks varied from 14 to 20, with a mean of 16.54 and a standard desviaton (SD) of 1.29 (Figure 2, parts D, E, and F; Table 1). Both sex chromosomes (X and Y) always presented C-bands but with different sizes (Figure 2E). Despite this variation, there was no clear difference in the number of chromosomes with C-bands among populations of the same localities but with different habitats (e.g., sylvatic and domestic populations from Jamach´Uma, Bolivia). Within group 1, the similar size and shape of the 10 chromosomal pairs made it very difficult to identify each pair. The C-heterochromatin content varied from 46% to 56% of the autosomal complement because of the heterochromatin polymorphism already mentioned. The mean haploid DNA content of all Andean specimens (12 insects) measured by flow cytometry was 1.825 + 0.149 pg (Table 3).

**Figure 3:**
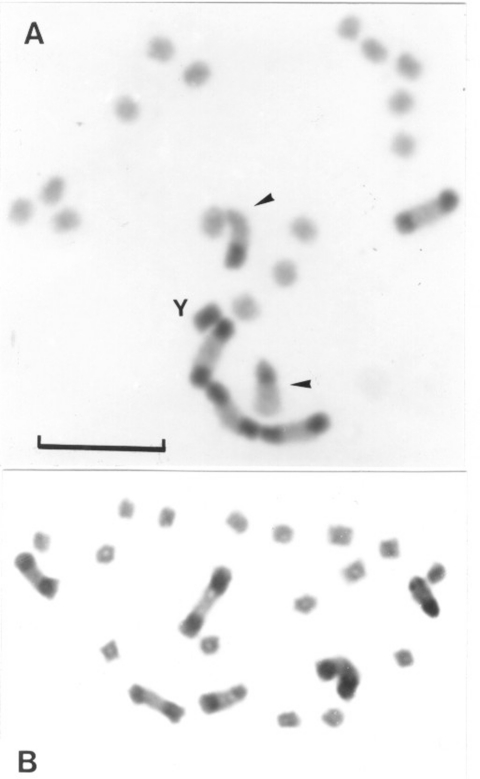
Gonial mitotic prometaphases in male (A) and female (B) specimens of *Triatoma infestans* from non-Andean regions. Scale bar = 10 μm. A: Most common C-banding pattern detected in non-Andean region (BB BB AA). This pattern is constituted by four autosomes with a C-block in both chromosomal ends (B morph) and two chromosomes with a C-block in only one telomere (A morph) indicated by arrowheads. The Y chromosome appears C-heterochromatic. The other 14 autosomes and the X chromosome are C-negative (euchromatic). B: Females only have C-bands in the autosomes; sex chromosomes (XX) are euchromatic and indistinguishable from autosomes without heterochromatin.

Group 2 includes specimens from all non-Andean populations, whose origins comprise three biogeographic regions: Chaco (Bolivian and Paraguayan Boreal Chaco, and Austral Chaco of Argentina), Pampeana (Uruguay), and Caatinga (Brazil) (Figure 2A, B, C; Figure 3). The number of autosomes with C-bands varied from four to seven chromosomes (mean 5.93 + 0.45) (Table 1), but almost all of the 139 insects presented six C-heterochromatic autosomes (86.33%). In this group, the three first autosomal C-heterochromatic pairs were identified, based on size differences and meiotic configurations. The karyotype described in previous reports, BB BB AA, was by far the most frequent (Table 2 and Figure 3A). The Y chromosome always exhibited C-blocks, whereas the X chromosome did not show any C-banding (Figure 3A). The 30 specimens in this group measured by flow cytometry had a mean of 1.401 + 0.111 pg of DNA per haploid nucleus (Table 3) in which the C-heterochromatin ranges from 24% to 30% of the total autosomal length.

Table 1 shows the number of C-heterochromatic autosomes in all samples studied. Table 2 details the C-banding patterns observed within non-Andean populations (group 2). The samples farthest away from the Andean region of Bolivia and Peru, e.g., the Brazilian Caatinga population, were the most homogeneous, almost always exhibiting the same C-karyomorphs (BB BB AA). By contrast, the population from the austral Chaco region of Argentina appeared quite variable, both in the number of C-banded autosomes as well as in the karyomorphs observed (Table 2). In the Andean population, we were unable to identify each chromosomal pair because of the similar size and shape of the autosomes.

Table 3 summarizes the haploid DNA content (expressed in picograms) observed in different populations of *T. infestans*. When group samples were compared, a reduction of 30% from Andean to non-Andean populations was detected. When the Jamach´Uma Domestic sample (Andean Bolivia) was compared with the dark morph population (non–Andean Bolivia) (Table 3), the non-Andean population had 40% less haploid DNA content.

### Analysis of Experimental Progeny between Andean and Non-Andean Populations

**Figure 4:**
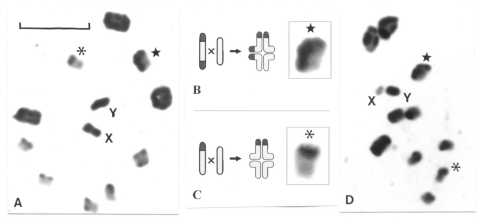
Meiotic pairing in the experimental male hybrid progeny between Andean and non-Andean specimens of *Triatoma infestans*. Scale bar = 10 μm. A: First meiotic metaphase in an insect obtained by crossing a female from Andean region (with C-banded X chromosomes) with a male from non-Andean region. As expected, both sex chromosomes are heterochromatic. As observed in normal specimens, the ten bivalents form a ring with the univalent sex chromosomes in the middle. Chromosome pairing was normal even between chromosomes with great heterochromatic differences. B: Selected bivalent with a diagram of its mitotic and meiotic configuration. As generally observed in hemipteran insects, only a single chiasma is represented. Chromosomes involved in the pairing have different C-patterns: one has C-bands in both chromosomal ends (B-morph); the other one is completely euchromatic (C-morph); the resulting bivalent is asymmetric. C: Selected bivalent with a diagram of its mitotic and meiotic configuration. Chromosomes involved have different C-patterns: one has C-bands in only one chromosomal end (A-morph) while the other one is completely euchromatic (C-morph); the resulting bivalent is also asymmetric. D: First meiotic metaphase in an insect obtained by crossing a female from non-Andean region (with euchromatic X chromosomes) with a male from Andean region. As expected, the X chromosome appears euchromatic in the hybrid. Chromosome pairing was completely normal even between chromosomes with great heterochromatic differences.

The meiotic behavior in the hybrids was apparently normal. A complete meiotic pairing was observed between the autosomes, and univalents were not detected (Figure 4). Several asymetric bivalents were clearly observed, formed by one chromosome with C-heterochromatin (one or two C-blocks) and another without C-heterochromatin (Figure 4). We could not detect any alteration in the form of the spermatids and spermatozoids. Moreover, the developmental cycle and fertility of the progeny did not show differences when compared to those of the parental generation.

## Discussion

### Chromosomal Groups in *Triatoma Infestans*

Our data disclosed two chromosomal groups in *T. infestans* here named Andean (Bolivian and Peruvian Andean samples) and non-Andean (samples from Argentina, Paraguay, Brazil, Uruguay, and Bolivian Chaco). These groups seem discrete and restricted to particular geographic areas; intermediate forms were not detected (Figure 1). These groups may be recognized by using three criteria: 1) the number of C-banded autosomes, 2) the C-banding on the X sex chromosome, and 3) the DNA content (Figures 2 and 3, Tables 1 and 2). The Andean specimens exhibited consistently more C-banded autosomes (14–20 autosomes) than non-Andean ones (4–7 autosomes); the Andean specimens showed a C-heterochromatic block in the X chromosome, which was absent in the non-Andean specimens, and all of them contained more DNA per cell (approximately 30% more) than did non-Andean specimens (Table 3).

### Taxonomic Status of *T. Infestans* Populations

Previous studies (*8*) suggested that heterochromatin could act as a fertility barrier in Triatominae by inhibiting meiotic pairing between chromosomes with different quantities of heteropyknotic regions. However, our analysis of experimental male progeny between both chromosomal groups (F1), where the chromosome pairing takes place without any apparent disturbance (Figure 4), shows that heterochromatin is not a postmating reproductive barrier, at least in *T. infestans*. Moreover, the subsequent developmental cycle and F1 fertility showed no difference with the parental generations (data not shown). Additional evidence for low level of divergence between these populations has been provided by other genetic techniques. Nei’s standard genetic distance between Andean and non-Andean populations based on allozyme frequencies was low, generally under 0.050 (*7*), and the DNA sequence comparison of a 412-bp fragment of the mitochondrial cytochrome B gene showed only three different nucleotide sites (*14*). At ribosomal DNA level, only 2 transversions and 4 insertions were found among the 459-bp-long ITS-2 (second internal transcribed spacer) between populations from Bolivia (Andean) and Paraguay (Chaco) (*15*). These data suggest that the genetic variation in the two groups of *T. infestans*, despite their strong chromosomal and DNA content differences, could be attributable to intraspecies variation.

### Biologic Significance of Heterochromatin Variation

Eukaryotic genomic DNA contains highly repetitive sequences, the relative amounts of which can differ markedly at population and interspecies levels. Many of the changes in genome size can be attributed to variation in the abundance of these repetitive sequences, rather than to large differences in the nonrepetitive fraction of unique DNA (coding sequences included). C-heterochromatin, revealed by C-banding, consists largely of highly repetitive simple DNA sequences (satellite DNA) and has long been regarded as inert or transcriptionally inactive. However, an extensive literature describes possible adaptive functions and effects of heterochromatin (*16*). An important and widespread effect of heterochromatin in germ cells both of plants and animals is its influence on the number and distribution of chiasmata. In most organisms, including *T. infestans* (*13*), the chiasmata either do not form, or form less frequently, in the euchromatic regions adjacent to the heterochromatin segments. Each heterochromatic block, through its chiasma displacement effect, can keep in its proximity certain favorable allele combinations of different genes (“coadapted gene pools”) (*17*). Deletion of C-block can release these zones, allowing recombination to occur and causing certain allele combinations to disappear, generate new ones, or both and as a consequence, influence the adaptability of the individual insect.

Variation in total DNA and heterochromatin contents has also been related to changes in biologic parameters, such as total cell volume, development rate, and body size (*18*). *T. infestans* specimens from Bolivia are indeed larger than those from Uruguay (*7*) or Brazil (*19*), suggesting that heterochromatin amounts could be related to morphologic parameters, and as a consequence, be the target of selective pressures (*18*).

### Origin of *T. infestans*

Based mainly on the existence of sylvatic populations in the Cochabamba valleys of Bolivia, several authors (*3*,*6*,*20*) have suggested that *T. infestans* originated in these Andean valleys. On the other hand, Carcavallo et al. (*21*) suggested that the origin of this species was in the dry subtropical forest from the South of Bolivia and Paraguay and the North of Argentina. This latter hypothesis was based on the discovery of sylvatic melanic forms of *T. infestans* (“dark morph”) in the Bolivian Chaco (*22*). However, the proposal of the dark morph as the original *T. infestans* population was not supported by body size measurements (*23*), antennal sensilla patterns (*24*), or isoenzymatic and mitochondrial data (*14*). Furthermore, cytogenetic results indicated that in dark morph specimens heterochromatin is restricted to three autosomal pairs (*25* and Table 2) and low DNA content (Table 3), suggesting their close relationship with our non-Andean chromosomal group. All these evidences strongly suggest that the dark morphs share a common origin with domestic non-Andean *T. infestans* and that they are not the original population, as suggested by Carcavallo et al. (*21*).

### Domestication Process

Despite some controversy about the origin of *T. infestans*, researchers generally agree that the adaptation of this species to human dwellings began in the Andean regions of Bolivia. There, sylvatic *T. infestans* is found in rock piles associated with small mammals such as wild guinea pigs (*Galea musteloides*) (*4*). Archaeological findings and historical reconstruction suggest that the domestication process occurred in pre-Colombian times, approximately 3,500 years ago (*6*), associated with the early settlements of pre-Incaic groups and the domestication of wild rodents for human food. The idea of a discrete Bolivian origin for domestic *T. infestans* is also supported by isoenzymatic studies (*7*,*26*). Hence, from Bolivia, domestic *T. infestans* spread over a major portion of South America.

### Geographic Spread of *T. infestans* in South America

*T. infestans* does not fly over long distances and depends mainly on its vertebrate hosts for dispersal; thus, its geographic expansion was most probably associated with human migrations. The settlement of pre-Incaic and Incaic tribes and their spread over substantial Andean regions could be the first series of events allowing passive dispersal of *T. infestans*. However, most of the dispersal of this species appears to have been associated with post-Colombian economic migrations in South America, particularly during the last 100–150 years (*6*). In Uruguay for example, *T. infestans* appears to have reached some southern communities along the River Plate by 1865 (*27*), but it was unknown in northern departments of Uruguay until the early 1900s, when it was apparently imported from southern Brazil by human migrations (*28*). This species also seems to have spread across the Sao Francisco River in Bahia during the early 1970s (*29*), arriving in the northeastern Brazilian states in the early 1980s (*30*). This rapid and recent geographic expansion of *T. infestans* from Andean countries to the south of the Neotropical region is supported by its relatively low genetic variability, as measured by isoenzymes (*7*,*26*) and mitochondrial (*14*) and ribosomal DNA sequencing data (*15*,*31*).

### Origin and Spread of Chromosomal Groups

#### Andean Dispersal

In light of the historical context mentioned above, *T. infestans* was originally a sylvatic species with large quantities of heterochromatin distributed in most of its chromosomal pairs (autosomes and sex chromosomes). This cytogenetic attribute was not deeply affected during the first phase of its geographic expansion throughout the Andean region of Bolivia and Peru. The domestic specimens in this region constituted an extended population cytogenetically similar to their putative sylvatic original population in central Bolivia (Tables 1 and 3, Figure 2D, E, and F).

### Non-Andean Dispersal

This dispersion in non-Andean regions involved *T. infestans* insects with a substantial loss of heterochromatic regions. This reduction is the main cause of the decrease in the DNA size of these insects. Although the mechanisms involved in this heterochromatin loss and DNA size reduction are unknown, several processes have been proposed in other organisms, such as unequal exchange and spontaneous deletion in nonessential DNA (*16*,*32*). Non-Andean populations of *T. infestans* could have been established first by one or a few founders that eventually lost part of their heterochromatin by random genetic drift. This kind of founder effect seems to play an important role in the genetic structure of *T. infestans* populations, as has been suggested by isoenzyme analysis (*7*,*26*,*33*). Moreover, the striking similarity among the C-banding patterns found in the non-Andean regions (Table 3), restricted to three heterochromatic pairs, suggests that the event of heterochromatin decrease may have taken place just once in the evolutionary history of *T. infestans*. This finding would imply that current populations of this insect outside Andean regions of Bolivia and Peru all derived from a single group of insects that were restricted to a particular region. Since Austral Chaco *T. infestans* in Argentina have the more variable C-banding patterns of the species from all the non-Andean areas (Table 1) and are geographically close to the Andean region, Austral Chaco was probably the primary focus of dispersal into the non-Andean region. The subsequent dispersion to other regions seems to have produced populations more homogeneous, in terms of number and localization of heterochromatic regions. Populations of recent colonization, such as those of Brazil and Uruguay, seem to have evolved towards the most common complement with three pairs of C-banded autosomes and a BB BB AA pattern (Figure 3A). In these populations, this karyotype is by far the most frequent and is the only one observed in the most recently colonized zones such as the Piaui state in Brazil (Table 1).

### Genomic Changes and Adaptive Processes

Genomic differentiation between both chromosomal groups is likely to be a reflection of both random drift and habitat adaptation. The novel genomic architecture of non-Andean group could have been triggered by a founder event. However, the success of these new small-genome insects is likely associated with adaptation to a new environment. One of the most noticeable differences in the domestic habitats of these groups is the altitude: Andean samples came from geographic regions generally above 1,800 m, whereas non-Andean populations were mainly from localities below 500 m (Table 1). Based on this geographic separation, our working hypothesis is that heterochromatin variation is a reflection of adaptive genomic changes that contribute to the ability of *T. infestans* to survive and reproduce in environments with different altitudes. According to this hypothesis, large-genome populations would be better adapted to Andean (highland) domiciles, while populations with small genomes would do better in non-Andean (lowland) houses. As a consequence, the success and spreading of each chromosomal group into Andean and non-Andean regions may indicate a better adaptation to the different selective pressures of its environment. A positive correlation between chromosome number and heterochromatin content with altitude has been described in other organisms (*34*,*35*). Nevertheless, other possible environmental factors or climatic variables associated with Andean and non-Andean habitats should not be discarded.

The inability to detect both chromosomal groups in a same region may also suggest a possible competition between them. The success of one chromosomal group with respect to the other would then depend on altitude. However, large-genome insects would be able to colonize lowlands, and small-genome insects would be able to colonize highlands.

This suggestion would explain the colonization by small-genome *T. infestans* of Argentina highlands (as we observed in the Anillaco sample). According to our altitude hypothesis, the Anillaco region should be a primary focus of colonization by *T. infestans* (small genomes), not previously colonized by large-genome insects. The analysis of very close locations with different altitudes in southern Bolivia and northern Argentina would contribute to testing our hypothesis that DNA content reduction reflects adaptive genomic changes related to altitude.

The adaptation of small genome insects to non-Andean domiciles could also be related to a loss in their capacity to return to sylvatic habitats. In non-Andean regions, *T. infestans* does not exhibit sylvatic foci, with the exception of atypical dark morph and melanosoma melanic variants (*14*,*22*). These facts could suggest that small-genome insects are unable to adapt to non-Andean sylvatic environments, unless they undergo new genetic changes that influence morphologic parameters.

In summary, we proposed that the genome size decrease observed in *T. infestans* was a successful change as it underwent adaptation to domiciles located in non-Andean lowland regions. However, the founder event generating this genomic variant could have also implied some loss of variability in particular loci. Greater domestic dependence, the inability to return to sylvatic ecotopes, and a certain degree of reduced variability could contribute to making these insects more susceptible to control campaigns, as observed in Uruguay, Chile, and Brazil. In future studies, socioeconomic, environmental, and operational issues also have to be taken into account so that the influence of vector genetic changes in control strategies can be evaluated. Furthermore, the existence of two alopatric groups in *T. infestans* with notable genomic differences is an important feature that have to be considered in evaluating vector control campaigns as well as in selecting the insect used in any genetic studies, including genome sequencing projects.
